# Enhanced Photocatalytic H_2_ Evolution over ZnIn_2_S_4_ Flower-Like Microspheres Doped with Black Phosphorus Quantum Dots

**DOI:** 10.3390/nano9091266

**Published:** 2019-09-05

**Authors:** Xiaoying Pan, Chaoqun Shang, Zhihong Chen, Mingliang Jin, Yongguang Zhang, Zhang Zhang, Xin Wang, Guofu Zhou

**Affiliations:** 1 National Center for International Research on Green Optoelectronics, South China Academy of Advanced Optoelectronics, South China Normal University, Guangzhou 510006, China; 2 International Academy of Optoelectronics at Zhaoqing, South China Normal University, Zhaoqing 526000, China; 3 Key Laboratory for Water Quality and Conservation of the Pearl River Delta, Ministry of Education, Institute of Environmental Research at Greater Bay, Guangzhou University, Guangzhou 510006, China

**Keywords:** ZnIn_2_S_4_, black phosphorus quantum dots, photocatalyst, hydrogen production

## Abstract

In this work, black phosphorus quantum dots (BPQDs) were decorated on hexagonal ZnIn_2_S_4_ flower-like microspheres to form zero-dimensional/two-dimensional (0D/2D) structures. Interface interactions between the BPQDs and ZnIn_2_S_4_ resulted in optimum effective charge transfer, thereby improving the photocatalytic performance of the material. Thus, the 0.2% BPQD–ZnIn_2_S_4_ sample showed 30% higher H_2_ evolution rates compared to pure ZnIn_2_S_4_. This study provides a simple route for the synthesis of photocatalysts. The results obtained herein can pave the way for designing effective catalysts for solar-to-chemical energy conversion and feasible approaches to obtain cheap, clean, and efficient photocatalysts.

## 1. Introduction

H_2_ is regarded as the most promising energy vector in the 21st century. Photocatalytic H_2_ evolution from water via solar irradiation is an attractive and sustainable solution for addressing the energy supply issue [1,2,3]. Since the photocatalytic splitting of water into H_2_ over TiO_2_ under solar irradiation was first discovered by Fujishima in 1972 [4], this reaction received broad attention. Thus, numerous efforts were made to develop non-toxic, clean, and efficient semiconductor photocatalysts. In this sense, a series of photocatalysts with enhanced photocatalytic H_2_ evolution activity such as CdS [5,6,7] and graphitic carbon nitride (g-C_3_N_4_) [8,9,10,11], among others, were developed so far.

Metal sulfides attract much attention owing to their suitable band position and strong visible-light absorption characteristics [12]. Among the ternary metal sulfides, chalcogenide ZnIn_2_S_4_ combines a suitable band gap, high chemical stability, and excellent photocatalytic activity, making it a suitable photocatalyst [13,14,15]. However, ZnIn_2_S_4_ suffers from a quick recombination of photogenerated electron–hole pairs and low migration ability for the photoexcited charge carriers. These are, in fact, the fundamental drawbacks of most semiconductors [16]. In order to optimize the photocatalytic performance of these materials, much effort was made for studying preparation methods leading to suitable particle size, structure, and morphology [17,18,19]. In addition, the photocatalytic activity depends significantly on the migration of the electron–hole pairs from the conduction band (CB) to the valence band (VB). Thus, the photocatalytic performance of ZnIn_2_S_4_ can be mainly improved by expanding its light absorption region and by eliminating the recombination centers of photoinduced electrons and holes. In this sense, numerous strategies such as metal doping [20,21], introduction of carbon quantum dots [13,22], and incorporation of other semiconductor materials [23,24,25,26] were used. However, photocorrosion and limited solar light utilization remain unsolved [22].

Black phosphorus (BP) is a two-dimensional (2D) material that attracted much attention in recent years owing to its excellent photoelectric properties (e.g., tunable bandgap and high charge carrier mobility) [27,28,29,30,31,32,33]. Lee’s group [34] prepared a hybrid BP@TiO_2_ material with enhanced photocatalytic performance (e.g., dye degradation and antibacterial activity) under light irradiation compared to pure BP. Zhu and co-workers [35] prepared 2D hybrid BP/WS_2_ with significantly higher H_2_ evolution rates compared to pure BP and WS_2_. In addition, this material released H_2_ under near-infrared (NIR) irradiation. Zhang [36] prepared, for the first time, black phosphorus quantum dots (BPQDs) and successfully used them in flexible memory devices. These memory devices showed a flash memory effect with high on/off current ratios and good stability. After this pioneering discovery, much attention was paid to this size-dependent material. More recently, Kong and co-workers [37] reported on BPQDs as co-catalysts of g-C_3_N_4_ for visible-light-driven photocatalytic H_2_ generation. Despite this broad application range, BPQDs are air- and water-sensitive, which limits their applications [38].

Herein, we decorated BPQDs on ZnIn_2_S_4_ (ZIS) and used the resulting material as a photocatalytic H_2_ production system for the first time. Under simulated sunlight irradiation, the prepared BPQD–ZIS hybrid photocatalysts showed significantly higher (ca. 1.32-fold) H_2_ generation rates compared to pure ZnIn_2_S_4_. The characterization results allowed us to propose reasonable mechanisms for this enhanced H_2_ production. The design of this new system can pave the way for new photocatalytic applications for these materials.

## 2. Experimental Section

### 2.1. Preparation

All chemicals were of analytical grade and used as received without further purification.

#### 2.1.1. Preparation of Hexagonal ZnIn_2_S_4_ Flower-Like Microspheres

In a typical procedure, 1.0 mmol of ZnCl_2_, 2.0 mmol of InCl_3_, and 6.0 mmol of thioacetamide (TAA) were dissolved in 80 mL of deionized water. The mixed solution was vigorously stirred for 30 min and then transferred to a 150-mL Teflon-lined autoclave. The autoclave was sealed, kept at 80 °C for 12 h, and then cooled to room temperature naturally. A yellow precipitate was filtered and washed with deionized water and absolute ethanol several times. Then, the solid was oven-dried at 60 °C overnight. The sample was denoted as ZIS.

#### 2.1.2. Preparation of a Dispersion of BPQDs

A dispersion of BPQDs was synthesized via solvent exfoliation with dimethylformamide (DMF). Firstly, 10 mg of bulk BP was added to 10 mL of DMF. The dispersion was sonicated for 6 h on an ultrasonic homogenizer (Scientz, JY92-II N, Ningbo, China) under ice cooling. After that, the dispersion was centrifuged at 5000 rpm for 20 min (to remove the non-exfoliated bulk BP) followed by centrifugation at 13,000 rpm for another 30 min to remove the BP flakes. The resulting material was a BPQD/DMF dispersion.

#### 2.1.3. Preparation of BPQD–ZIS Materials

BPQD–ZIS materials were prepared by adding 200 mg of ZIS to DMF, followed by ultrasonication of the mixture to form a homogeneous dispersion. Subsequently, the as-prepared BPQD/DMF dispersion was added to a ZIS dispersion and sonicated for 2 h, and the mixture was stirred overnight. The as-obtained powders were collected by high-speed centrifugation, washed with deionized water and ethanol thoroughly, and finally vacuum-dried at 40 °C overnight to obtain BPQD–ZIS. Several BPQD–ZIS materials with various weight ratios were prepared by adding different amounts of the BPQD dispersion to the ZIS dispersion.

### 2.2. Characterization

The morphology of the hybrid materials was characterized by field-emission scanning electron microscopy (SEM, FEI Quanta 250 FEG, FEI, Hillsboro, OR, USA). Transmission electron microscopy (TEM), high-resolution transmission electron microscopy (HR-TEM), and energy-dispersive spectrometry mapping (EDS mapping) images were obtained on a JEM-2100HR device (JEOL, Tokyo Japan). X-ray diffraction (XRD) patterns and X-ray photoelectron spectra (XPS) were obtained on BRUKER D8 ADVANCE (BRUKER, Karlsruhe, Germany) and Thermos SCIENTIFIC ESCALAB 250Xi devices(Thermos, Boston, MA, USA), respectively. Fourier-transform infrared (FT-IR) spectroscopy was conducted on a BRUKER VERTEX 70 device (BRUKER, Karlsruhe, Germany). Ultraviolet–visible light (UV–Vis) diffuse reflectance spectroscopy (DRS) was conducted on a METASH UV-8000A device (METASH, Shanghai, China), while the photoluminescence (PL) spectra were obtained on a GANGDONG F-380 fluorescence spectrophotometer (Gangdong, Tianjin, China).

### 2.3. Photoelectrochemical Measurements

Photoelectrochemical measurements of the obtained products were carried out on an electrochemical analyzer (CHI660E) in a standard three-electrode system using the prepared samples as the working electrodes with an active area of 1 cm^2^, a Pt wire as the counter electrode, and saturated Ag/AgCl as the reference electrode. An aqueous Na_2_SO_4_ (0.2 M) solution was used as the electrolyte. The electrochemical impedance spectra (EIS) were obtained by applying an alternate current amplitude of 5.0 mV in a frequency varying from 100 kHz to 0.01 Hz.

### 2.4. Photocatalytic H_2_ Evolution

Typically, 50 mg of catalyst powder was added to a cylinder reactor containing an aqueous solution (100 mL) containing 0.35 M Na_2_S and 0.25 M Na_2_SO_3_ and sealed with a rubber septum. Before the reaction, Ar purges were carried out through the solution to remove the O_2_ and N_2_ dissolved in the water. The reaction was carried out by irradiating the suspension with simulated sunlight with a 300-W Xe lamp (Perfect Light, PLS-SXE300, Beijing, China) equipped with an optical filter AM 1.5 (Perfect Light, Beijing, China). The gas produced over the catalyst was collected and analyzed by gas chromatography (GC, Shiweipuxin GC-7806, Shiweipx, Beijing, China).

## 3. Results and Discussion

### 3.1. Characterization of the Photocatalysts

**Figure 1:**
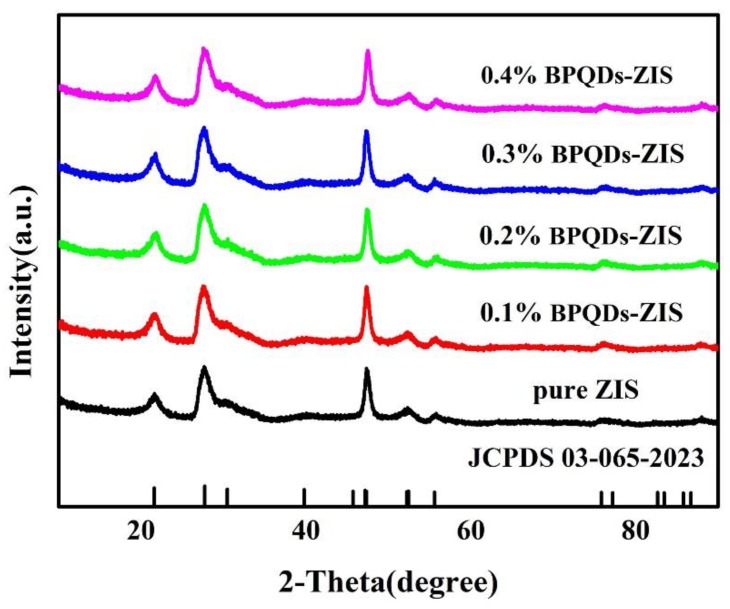
X-ray diffraction (XRD) patterns of pure ZnIn_2_S_4_ (ZIS) and black phosphorus quantum dot (BPQD)–ZIS hybrids.

The XRD patterns of the pure ZnIn_2_S_4_ and hybrid BPQD catalysts (BPQD loadings of 0.1, 0.2, 0.3, and 0.4 wt.%) are presented in Figure 1. Intense peaks were observed at 2θ values of 20.44, 28.47, 47.32, 52.61, 55.97, and 76.30°, and these peaks were ascribed to the (006), (102), (110), (022), (116), and (212) lattice planes of a hexagonal ZnIn_2_S_4_ phase (JCPDS card No. 03-065-2023) [39], respectively. These results indicated that the presence of BPQDs did not alter the crystal structure of ZnIn_2_S_4_. The low loadings of BPQDs prevented us from observing apparent peaks corresponding to BPQDs in the BPQD–ZIS samples.

**Figure 2:**
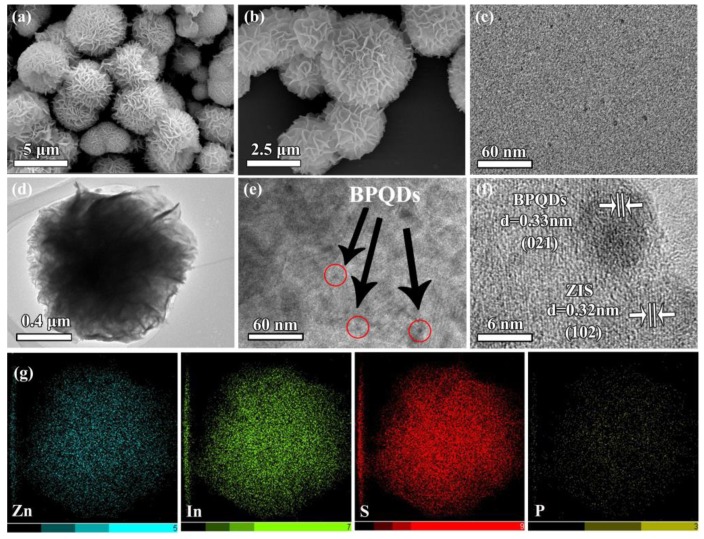
SEM images of ZnIn_2_S_4_ (**a**,**b**), TEM images of BPQDs (**c**) and BPQD–ZIS (**d**,**e**), high-resolution TEM (HRTEM) images of BPQD–ZIS (**f**), and elemental mapping of BPQD–ZIS (**g**).

The morphology of the prepared BPQD–ZIS was confirmed by SEM. As shown in Figure 2, pure ZnIn_2_S_4_ contained flower-like microspheres of ca. 2–5 μm in diameter. As revealed by TEM (Figure 2d), the flower-like microspheres of the synthesized BPQD–ZIS materials contained numerous ultrathin nanosheets. The presence of these nanosheets rendered ZnIn_2_S_4_ with a unique layered structure. Thus, the ZnIn_2_S_4_ flower-like microspheres increased the contact area for BPQD decoration. BPQDs were observed at the edges of the flower-like microspheres, revealing that BPQD-doped ZnIn_2_S_4_ hybrids were successfully synthesized. A lattice spacing of 0.32 nm was determined (Figure 2f) and assigned to the (102) facet of hexagonal ZnIn_2_S_4_ [15]. The inter-planar spacing of ca. 0.33 nm corresponded to the (021) facet of BPQDs [40] in the hybrid material. These results demonstrated that BPQDs were introduced into the ZnIn_2_S_4_ phase, generating a good contact between the BPQDs and ZnIn_2_S_4_ in the BPQD–ZIS hybrids. Elemental mapping of the BPQD–ZIS samples revealed the presence of P, Zn, In, and S, confirming that the BPQD–ZIS hybrids were successfully prepared.

**Figure 3:**
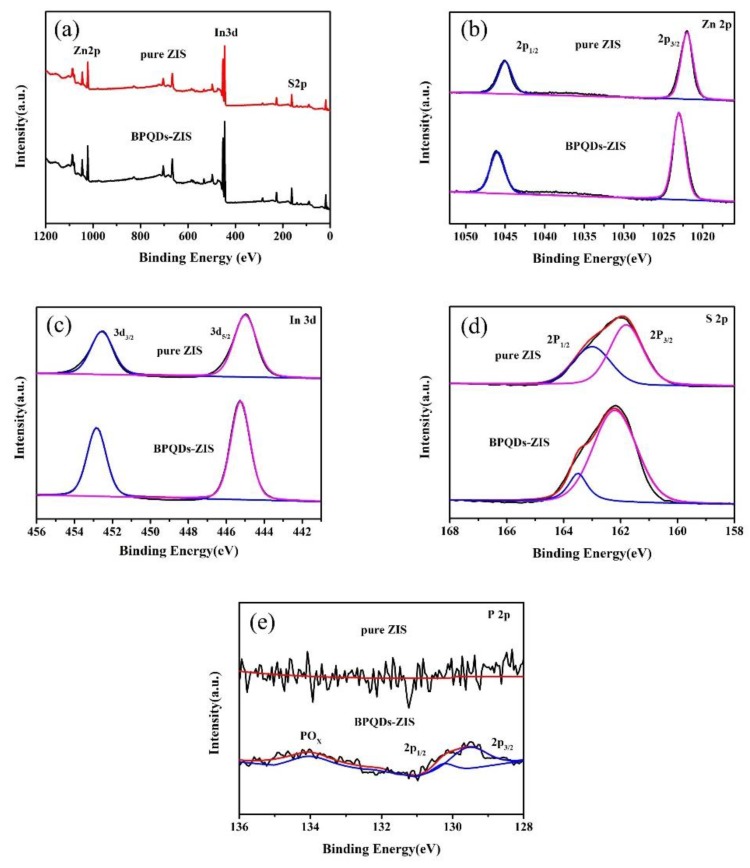
Full survey scanned X-ray photoelectron spectroscopy (XPS) spectra of ZnIn_2_S_4_ and BPQD–ZIS (**a**); Zn 2*p* (**b**), In 3*d* (**c**), S 2*p* (**d**), and P 2*p* (**e**) high-resolution XPS spectra of ZnIn_2_S_4_ and BPQD–ZIS.

To gain insight into the interfacial interaction between ZnIn_2_S_4_ and BPQDs, XPS measurements (Zn 2*p*, In 3*d*, S 2*p*, and P 2*p*) were carried out. The full survey scanned spectra (Figure 3a) of ZnIn_2_S_4_ and the BPQD–ZIS hybrids contained the Zn 2*p*, In 3*d*, and S 2*p* bands. The high-resolution XPS Zn 2*p* band of ZnIn_2_S_4_ (Figure 3b) contained two peaks at 1044.88 and 1022.34 eV, corresponding to the Zn 2*p*_1/2_ and Zn 2*p*_3/2_ transitions of Zn^2+^ species, respectively. The peaks at 452.61 and 445.1 eV (Figure 3c) corresponded to In 3*d*_3/2_ and In 3*d*_5/2_ transitions of In^3+^ species, respectively. The peaks at 163.18 and 161.82 eV (Figure 3d) corresponded to the S 2*p* band of ZnIn_2_S_4_, and were attributed to S 2*p*_1/2_ and S 2*p*_3/2_ transitions [39], respectively. However, the Zn 2*p*, In 3*d*, and S 2*p* bands shifted to higher binding energies for the BPQD–ZIS hybrid material compared to ZnIn_2_S_4_. The electronic coupling between ZnIn_2_S_4_ and BPQDs upon introduction of BPQDs can account for these shifts. Furthermore, as shown in Figure 3e, the three characteristic P *2p* peaks at ca. 129.8, 130.6, and 133.6 eV were observed for BPQDs [37] after curve-fitting, and these peaks corresponded to P 2*p*_3/2_, P 2*p*_1/2_, and oxidized phosphorus (POx), respectively. BP crystals are very stable in air. However, when BP is in the form of QDs, it becomes especially sensitive to water and oxygen. Since our experiments were carried out in air, BPQDs were unavoidably oxidized during the process. The high-resolution P 2*p* XPS spectra of the BPQD–ZIS samples further confirmed the formation of the BPQD–ZIS hybrid system.

**Figure 4:**
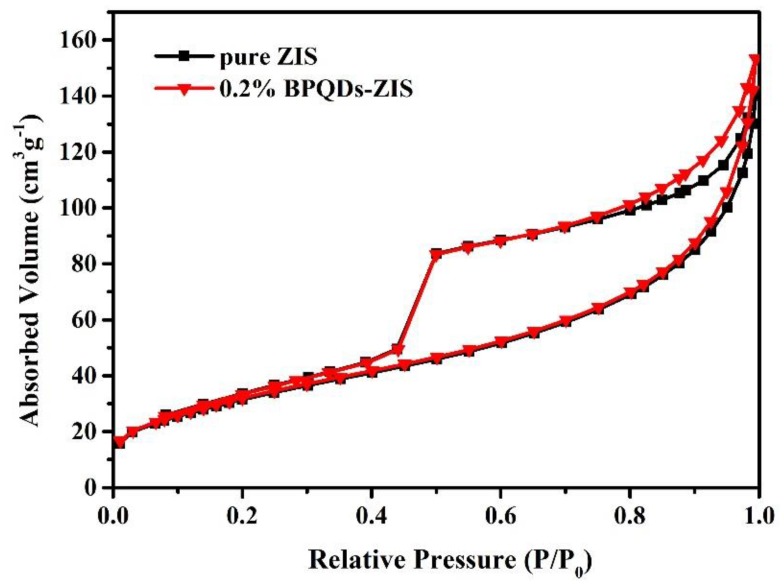
The nitrogen adsorption/desorption isotherm of pure ZIS and 0.2% BPQD–ZIS samples.

The Brunauer–Emmett–Teller (BET) specific surface area of pure ZIS and 0.2% BPQD–ZIS samples were investigated by N_2_ adsorption–desorption measurements. As can be clearly seen in Figure 4, the curved shape of the samples belonged to a type IV isotherm with hysteresis loops characteristic of a mesoporous structure. The BET specific surface areas of pure ZIS and 0.2% BPQD–ZIS were calculated to be 116.35 and 118.52 m^2^∙g^−1^, suggesting that the incorporation of BPQDs had negligible influence on the surface area of ZIS. The slight larger surface area of the 0.2% BPQD–ZIS sample may be due to testing errors.

**Figure 5:**
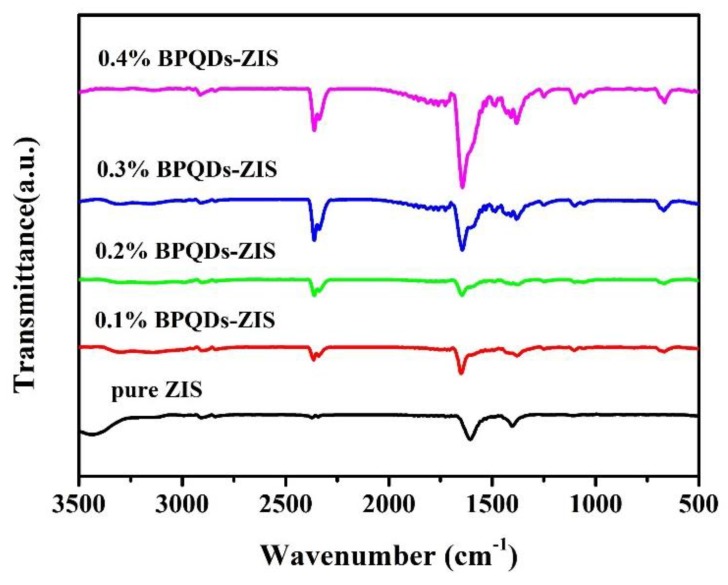
Fourier-transform infrared (FT-IR) spectra of ZnIn_2_S_4_ and the BPQD–ZIS samples.

FT-IR spectroscopy was conducted to further characterize the prepared samples and the results are shown in Figure 5. Pure ZIS and the BPQD–ZIS samples showed similar FT-IR bands. The peaks at about 1620 and 1385 cm^−1^ corresponded to the hydroxyl group and surface-absorbed water molecules, and the absorption peak found at 1100 cm^−1^ was assigned to the absorbed CO_2_. The characteristic peaks of BPQDs were not observed [13,41], while the peak located at around 640 cm^−1^ could be assigned to the stretching vibrations of In–S [42], which gradually enhanced with increased BPQDs. The reason for this phenomenon is that the incorporation of BPQDs may influence the stretching vibrations of In–S, resulting in increased peak intensity. These results further confirmed the contact between the BPQDs and ZnIn_2_S_4_ and the formation of BPQD–ZIS hybrids.

**Figure 6:**
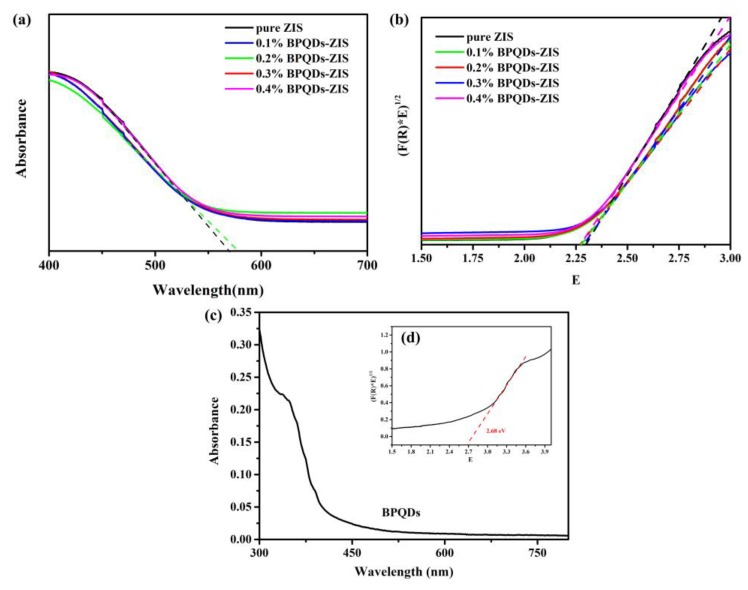
(**a**) Ultraviolet–visible light (UV–Vis) spectra of ZnIn_2_S_4_ and the BPQD–ZIS samples; (**b**) Tauc plots of ZnIn_2_S_4_ and the BPQD–ZIS samples; UV–Vis spectra (**c**) and the corresponding plot (**d**) of BPQDs.

DRS was used to gain insight into the light absorption properties of ZnIn_2_S_4_ and the hybrid BPQD–ZIS materials. As shown in Figure 6a, all samples showed nearly similar absorption spectra. The spectra of the hybrid BPQD–ZIS materials red-shifted, and this can be ascribed to absorption of light by the BPQDs.

In order to determine the band gap (*E*g) of the samples, the Kubelka–Munk function was used.

*A* = −lg(*R*),

*F*(*R*) = (1 − *R*)^2^/2*R*,

*E* = 1240/wavelength.

The results were obtained by plotting (*E* × *F*(*R*))^1/2^ with respect to *E*, where *R* is the reflectivity, and *A* is the absorbance. As displayed in Figure 6b, these samples had a similar band gap. After the incorporation of BPQDs, the band gap of the samples slightly narrowed, whereby the results of pure ZIS, 0.1% BPQD–ZIS, 0.2% BPQD–ZIS, 0.3% BPQD–ZIS, and 0.4% BPQD–ZIS were 2.29, 2.26, 2.25, 2.28, and 2.26 eV, respectively. In addition, the band gap of BPQDs was also measured to be 2.68 eV, as shown in Figure 6c,d.

### 3.2. Photocatalytic H_2_ Evolution Performance of the Photocatalysts

**Figure 7:**
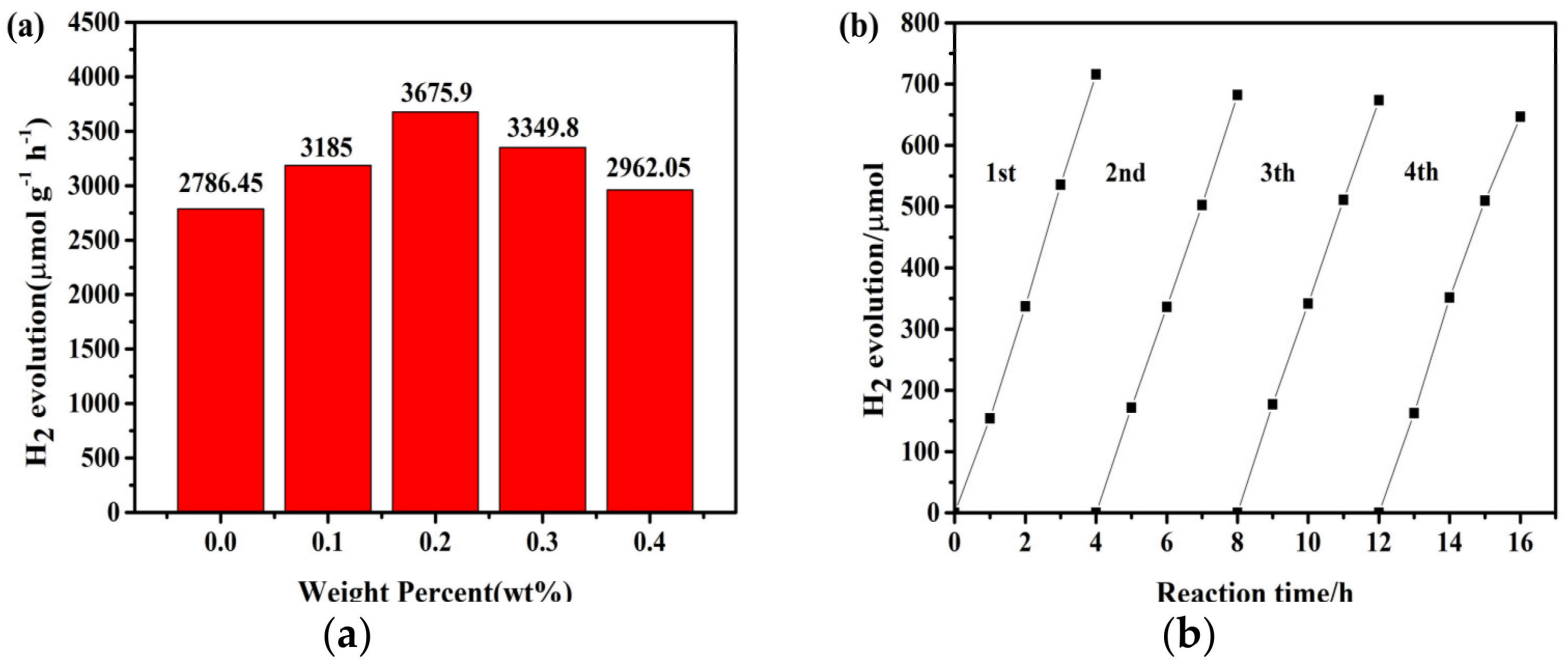
(**a**) H_2_ production during 4 h over ZnIn_2_S_4_ and BPQD–ZIS hybrids under light irradiation; (**b**) cycled runs for the photocatalytic H_2_ generation over 0.2% BPQD–ZIS under light irradiation

The photocatalytic H_2_ production activities of ZnIn_2_S_4_ doped with different loadings of BPQDs were determined under simulated sunlight irradiation. As shown in Figure 7a, pure ZnIn_2_S_4_ released H_2_ (ca. 2786.45 μmol∙g^−1^∙h^−1^) in aqueous solution (Na_2_S 0.35 M/Na_2_SO_3_ 0.25 M). This may be ascribed to the fast recombination of the photoinduced electron–hole pairs between the CB and VB of ZnIn_2_S_4_. Moreover, 0.2 wt.% was found to be the optimal BPQD loading, resulting in photocatalytic H_2_ rates 1.3 times higher (3675.9 μmol∙g^−1^∙h^−1^) compared to pure ZnIn_2_S_4_. The total yield of H_2_ increased significantly for the hybrid samples compared to pure ZnIn_2_S_4_. These results confirmed that decoration of BPQDs on ZnIn_2_S_4_ improved the photocatalytic activity of the materials. A further increase in the BPQD loading above 0.2 wt.% resulted in lower H_2_ production rates, probably because a fraction of the BPQDs acted as photo-recombination centers.

To confirm the positive effect of BPQDs in enhancing the photocatalytic H_2_ production activity of ZIS, the photocatalytic H_2_ production values of 0.2% BPQD–ZIS in this work were compared with the related literature, listed in Table 1. It can be clearly found that 0.2% BPQD–ZIS in this work showed superior photocatalytic performance to that of other quantum dots, implying that the incorporation BPQDs onto the ZIS surface may be a feasible route to improve its photocatalytic activity.

Recycling tests were carried out to evaluate the photostability of 0.2% BPQD–ZIS (Figure 7b). The yield of H_2_ remained relatively stable for four consecutive runs, indicating the stability of the BPQD–ZIS sample during the photocatalytic H_2_ production reaction.

**Figure 8:**
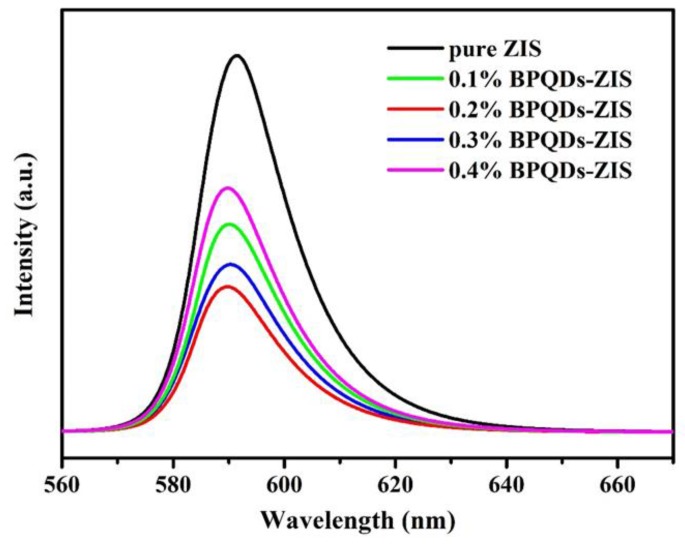
Photoluminescence (PL) spectra of pure ZnIn_2_S_4_ and BPQD–ZIS hybrids.

The charge recombination properties of the as-prepared samples were further confirmed by PL measurements. Normally, the PL intensity is related to the recombination of holes and electrons. The lower PL intensity corresponds to the lower recombination of the photogenerated electron–hole pairs. The PL spectra of all as-prepared samples are shown in Figure 8. All samples showed a similar PL peak and the intensity of BPQD–ZIS hybrids obviously decreased compared with that of pure ZIS. The weakest peak intensity suggested the recombination of photogenerated electron–hole pairs was efficiently suppressed in the 0.2% BPQD–ZIS. However, when the BPQD loading mass was more than 0.2 wt.%, an evidently increased PL emission intensity occurred, which might be ascribed to the excess BPQDs acting as photorecombination centers, leading to lower H_2_ production rates.

**Figure 9:**
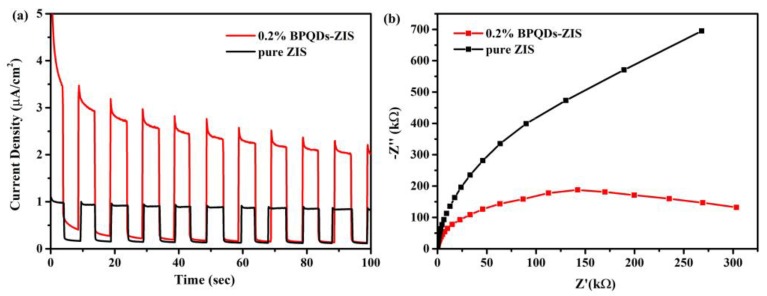
Transient photocurrent response (**a**) and electrochemical impedance spectra (**b**) of pure ZnIn_2_S_4_ and 0.2% BPQD–ZIS hybrids.

The transient photocurrent response and EIS were conducted to evaluate the charge separation and migration behavior. As shown in the Figure 9a, the pure ZIS exhibited a weak photocurrent response, owing to the fast photogenerated carrier recombination, while the photocurrent response of 0.2% BPQD–ZIS was much higher than that of the bare ZIS, which suggested that the charge recombination was remarkably inhibited. It can be clearly found in Figure 9b that the 0.2% BPQD–ZIS sample displayed a smaller semicircle than that of pure ZIS, suggesting that the charge transfer resistance can be efficiently reduced with the incorporation of BPQDs. Both the results of photocurrent and EIS experiments indicated that the incorporation of BPQDs onto the ZIS surface can facilitate charge carrier separation and transfer in the photocatalyst.

**Figure 10:**
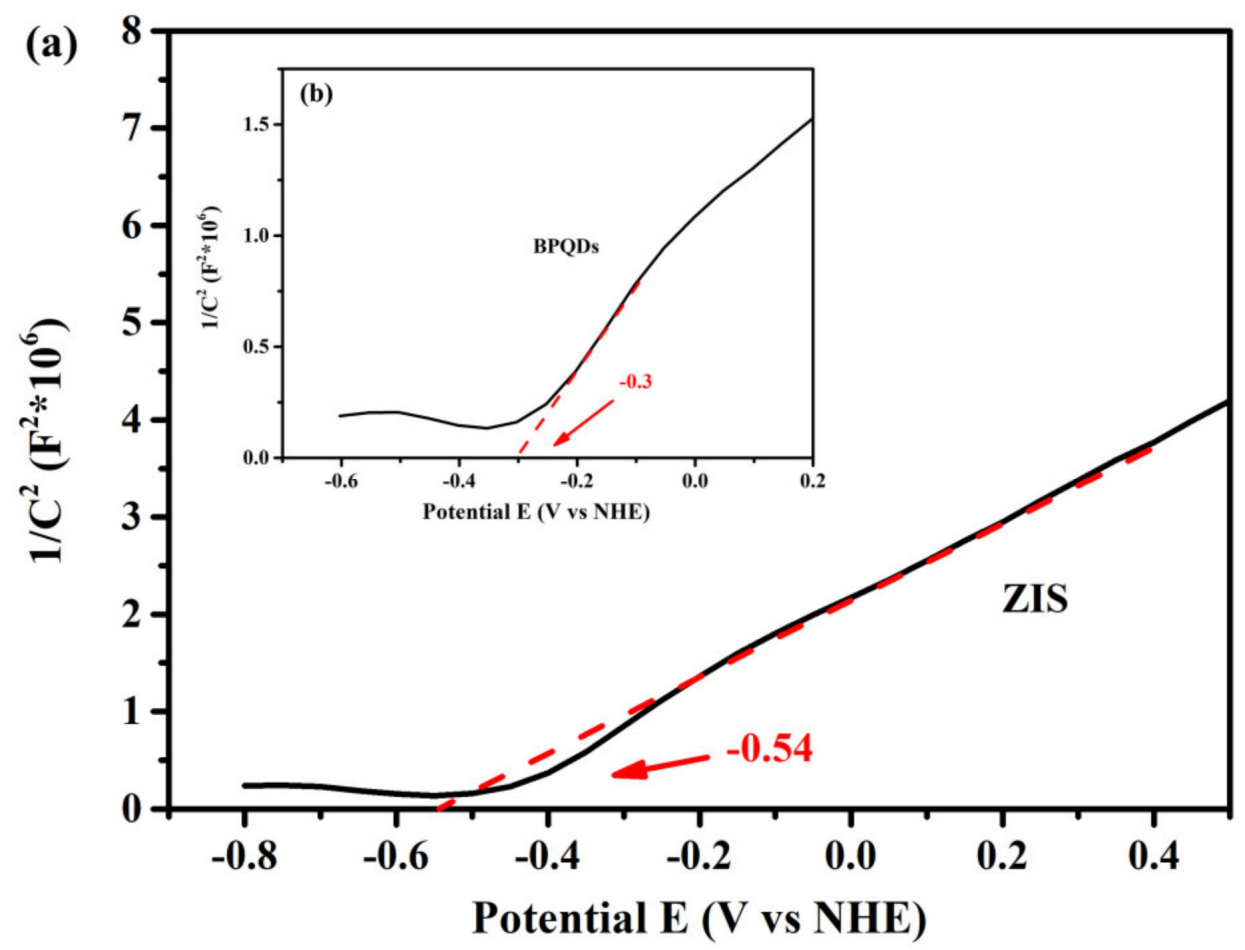
The Mott–Schottky plots of pure ZnIn_2_S_4_ (**a**) and BPQDs (**b**).

The Mott–Schottky (M–S) plots were conducted to determine the flat-band potential for pure ZIS and BPQDs. As seen from Figure 10, the positive slope of the M–S plots signified the type of ZIS and BPQDs as both n-type semiconductors, and, on the basis of the M–S equation, the flat-band potentials (*U*_fb_) for ZIS and BPQDs were determined as −0.54 and −0.3 V vs. NHE (Normal Hydrogen Electrode), respectively. Normally, the bottom of the conduction band (CB) position of an n-type semiconductor is 0.1 V more negative than the flat-band potential [47]. Therefore, the conduction band potentials (CB) of ZIS and BPQDs are approximately −0.64 and −0.4 V. Meanwhile, combining the DRS results and the equation *E*_VB_ = *E*_CB_ + *E*g, the values of the valence band potential (*E*_VB_) were determined as 1.65 and 2.28 V vs. NHE for ZIS and BPQDs, respectively.

### 3.3. Mechanism behind the Enhanced Photocatalytic Performance of the BPQD–ZIS Hybrids

**Scheme 1:**
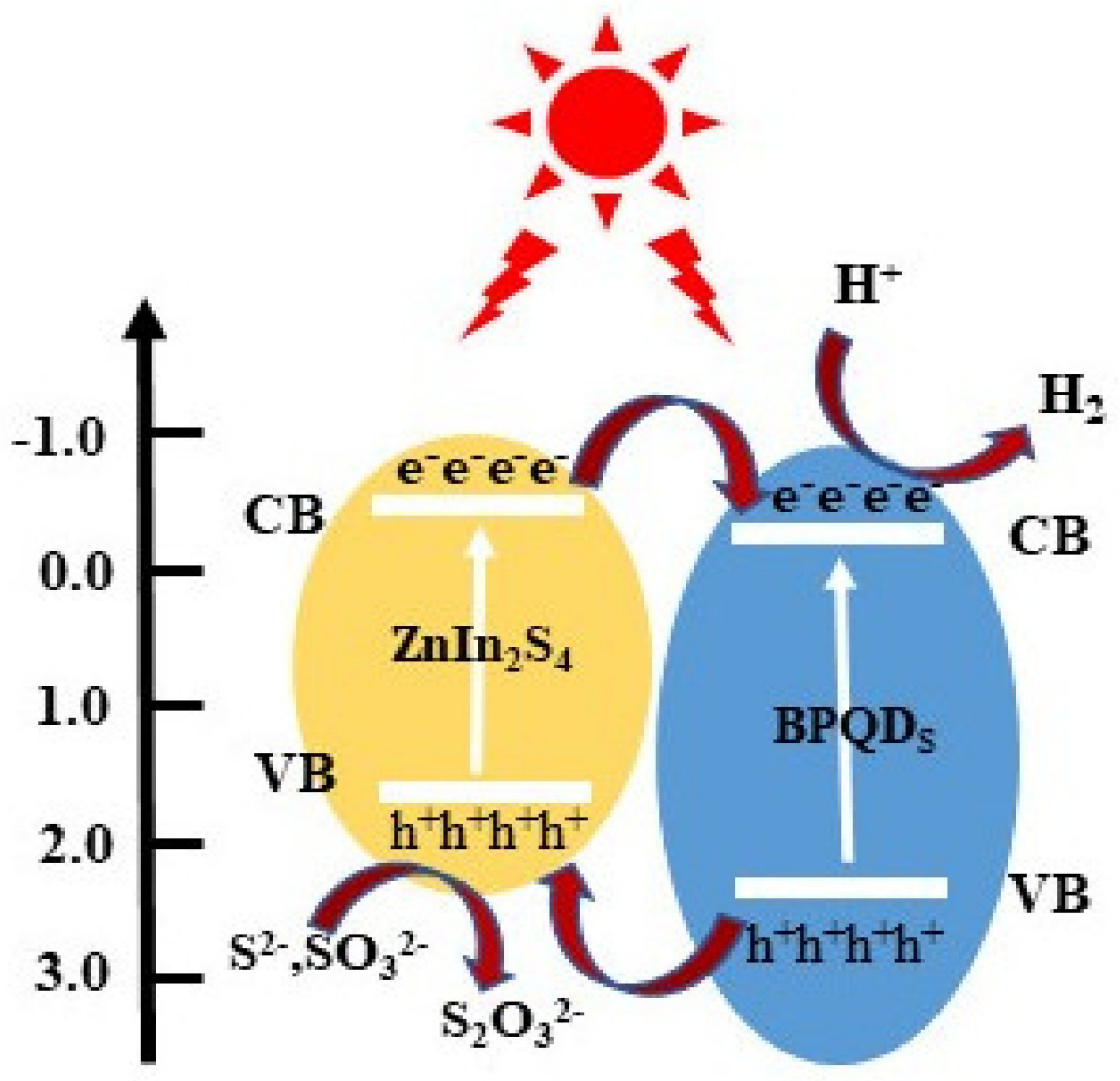
Schematic illustration of the degradation mechanism of the BPQD–ZIS hybrid.

Based on above analysis, we propose a mechanism to explain the BPQD–ZIS photocatalytic H_2_ evolution under light irradiation (Scheme 1). Under simulated sunlight irradiation, ZnIn_2_S_4_ is photoexcited and generates electrons and holes. The electrons are transfer to the CB, while the holes remain in the VB. Typically, these electrons and holes are recombined rather than participating in the photocatalytic process. In the presence of BPQDs, the photo-generated electrons can be transferred to the surface of BPQDs quickly because of the lower energy level of BPQDs. Meanwhile, the photo-generated holes can migrate to the VB of ZnIn_2_S_4_, achieving efficient separation of charges. Thus, the electrons on the surface of BPQDs can participate in the H_2_ evolution reaction, and the holes on the surface of ZnIn_2_S_4_ can finish the oxidation process.

## 4. Conclusions

In summary, we, for the first time, decorated BPQDs on layered flower-like ZnIn_2_S_4_ microspheres and used the resulting materials for photocatalytic H_2_ generation. XRD, FTIR, UV–Vis absorption, and XPS were used to study the physicochemical properties of the samples. These characterization results confirmed that BPQDs were embedded into the nanosheets of the flower-like ZnIn_2_S_4_ microspheres. The prepared BPQD–ZIS hybrids were quite stable during the photocatalytic H_2_ generation in an aqueous solution. The enhanced photocatalytic properties of these materials can be attributed to the extended optical absorption and efficient charge separation and transport characteristics upon addition of BPQDs. The 0.2% BPQD–ZIS sample showed an optimum H_2_ yield under simulated sunlight. This sample showed higher H_2_ production rates than pristine ZnIn_2_S_4_.

## Figures and Tables

**Table 1:** Comparation in some photocatalysts.

Photocatalyst	Sacrificial Reagent	Rate of Hydrogen Evolution (μmol·h^−1^·g^−1^)	References
BPQD–ZnIn_2_S_4_	0.25 M Na_2_SO_3_/0.35 M Na_2_S	3675.9	Our work
Carbon quantum dots (CQDs)/MoS_2_/ZnIn_2_S_4_	0.25 M Na_2_SO_3_/0.35 M Na_2_S	3000	[43]
Co/CQDs/ZnIn_2_S_4_	0.1 M triethanolamine	1757.5	[44]
NiS/CQDs/ZnIn_2_S_4_	10 vol.% triethanolamine	568	[45]
CdS QDs/ZnIn_2_S_4_	0.25 M Na_2_SO_3_/0.35 M Na_2_S	2145	[46]
